# Supplementary material for: Frontotemporal Lobar Degeneration Case with an N-Terminal TUBA4A Mutation Exhibits Reduced TUBA4A Levels in the Brain and TDP-43 Pathology
Source: Biomolecules. 2022 Mar 12;12(3):440. doi: 10.3390/biom12030440 (PMC8946841; doi:10.3390/biom12030440)
Supplement: Supplementary file 1 [file biomolecules-12-00440-s001.zip › biomolecules-1591148-supplementary.pdf]

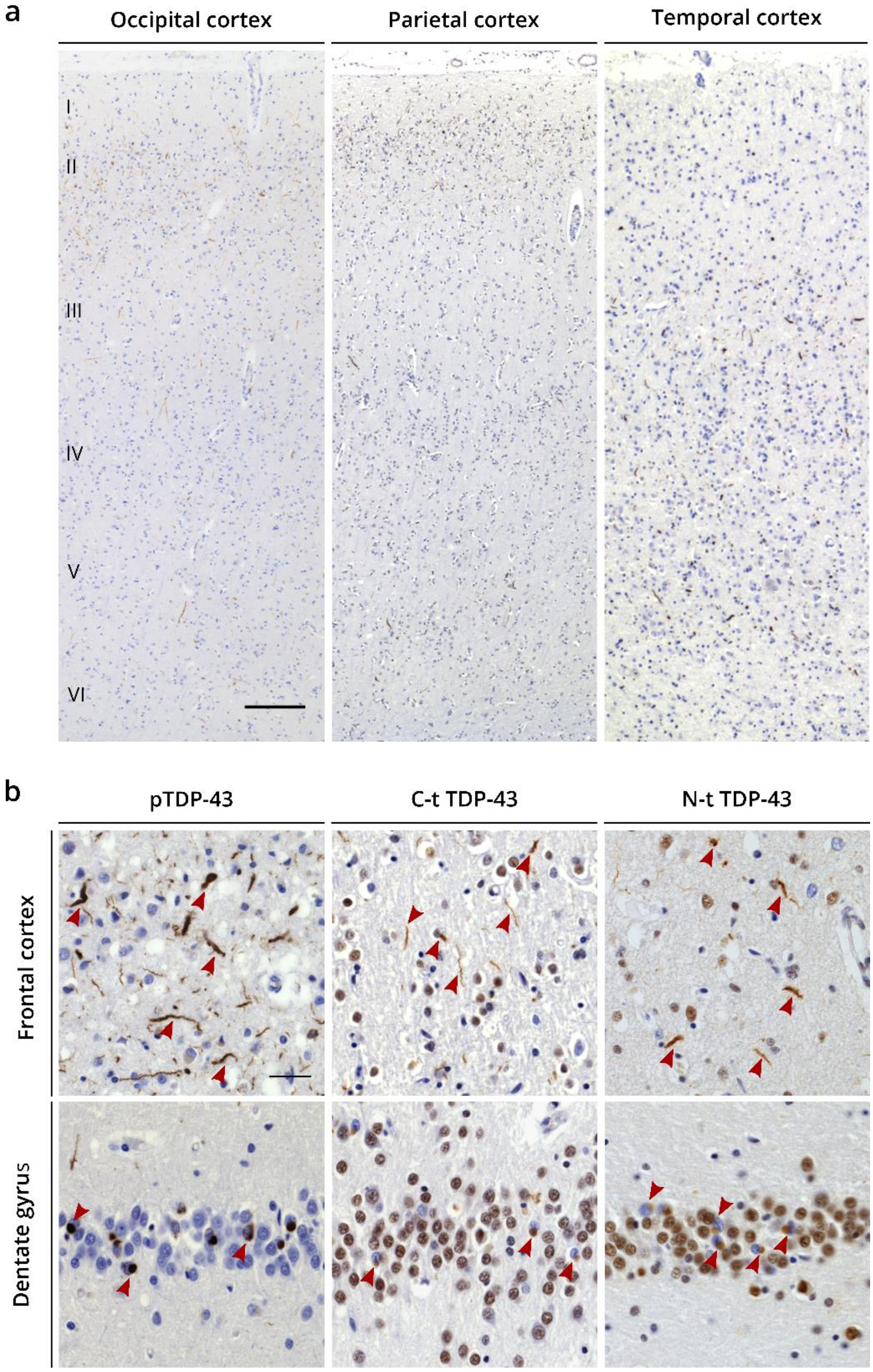

Supplementary Figure S1. TDP-43 pathology in the FTLD-TDP case with an R64Gfs*90 *TUBA4A* mutation. (a) pTDP-43 pathology is spread throughout all layers of the occipital and parietal cortex and the temporal cortex of the R64Gfs*90 *TUBA4A* mutation case. **(b)** pTDP-43 lesions in the dentate gyrus and frontal cortex (layer II) of the R64Gfs*90 *TUBA4A* mutation case are immunopositive for N-t TDP-43 and C-t TDP-43 antibodies (arrowheads). Scale bars represent 200 µm (a) and 15 µm (b).

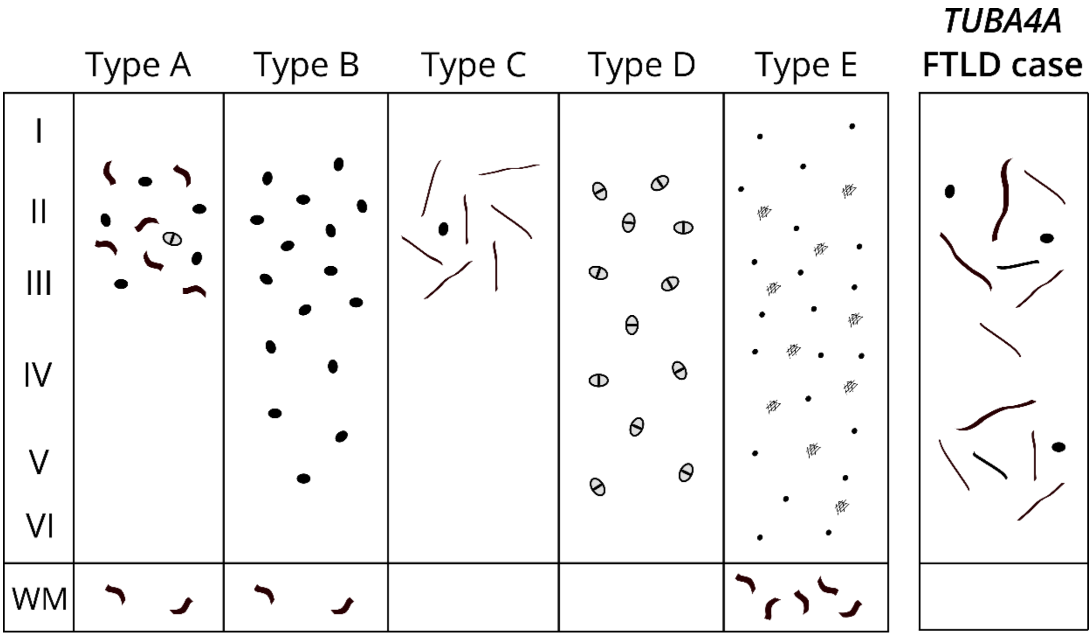

Supplementary Figure S2. Schematic overview of the FTLD-TDP pathology pattern. This scheme provides an overview of the FTLD-TDP types that have been described previously. The pattern of the R64Gfs*90 *TUBA4A* mutation case fits best with FTLD-TDP Type C. Pathological lesions mainly consisting of dystrophic neurites were observed throughout all layers of the cortex and were absent in the white matter.

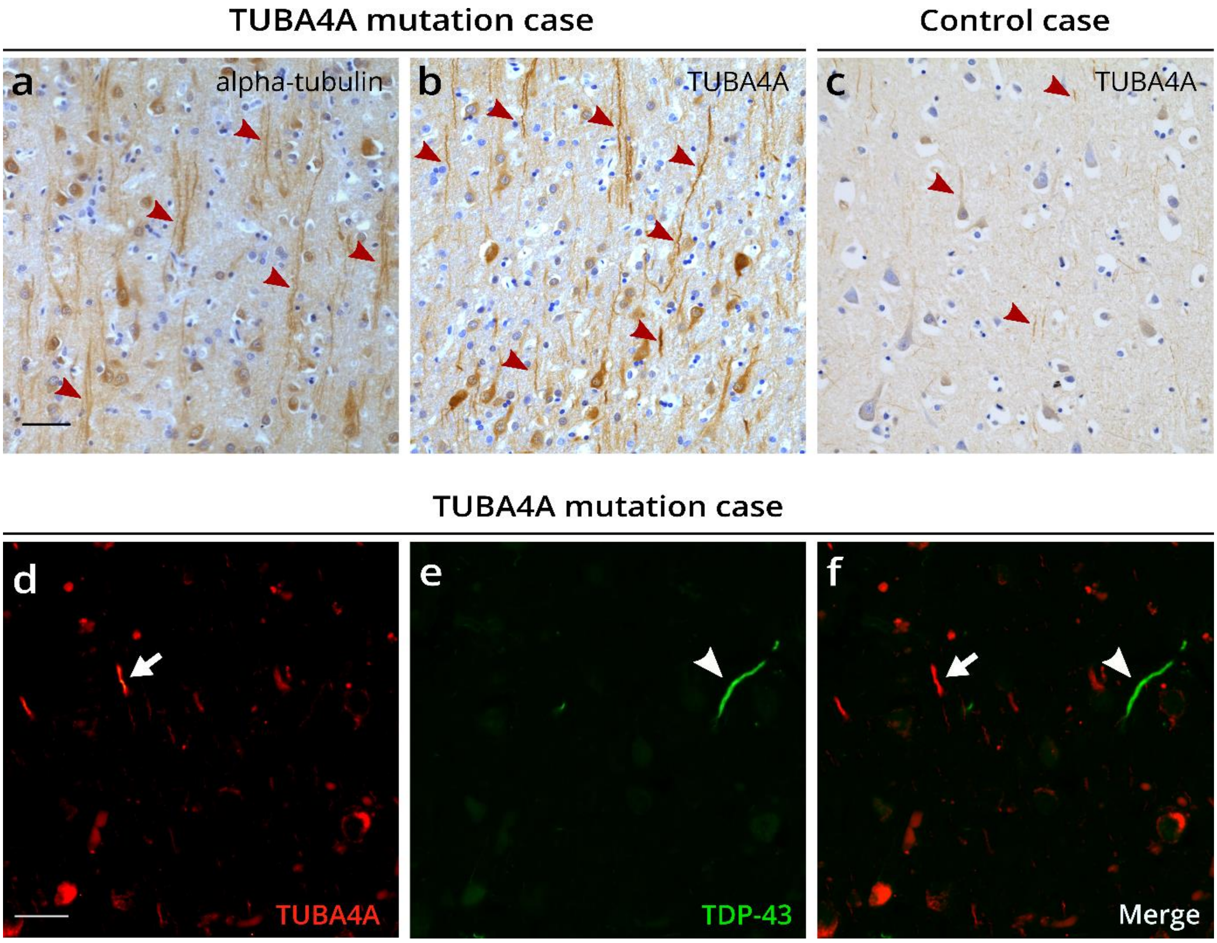

Supplementary Figure S3. TUBA4A immunohistochemical analysis. (a-c) Antibodies against alpha-tubulin (a) and TUBA4A (b) show a divergent staining in the R64Gfs*90 *TUBA4A* mutation case with abnormal kinking of apical dendrites (arrows in a, b) compared to control cases (arrows in c) in the frontal cortex (layer V). **(d-f)** TUBA4A thickened neurites (arrow) and TDP-43 dystrophic neurites (arrowhead) do not co-localize in the frontal cortex (layer V) of the R64Gfs*90 *TUBA4A* mutation case. Scale bars represent 50µm.

Supplementary Table S1. Overview of the autopsy cases used for indicated experiments. The table provides information regarding age (= age at death), sex, diagnosis, the presence of a genetic mutation, Braak NFT stage, AβMTL phase, Braak LBD stage, PMI (hours) and RIN values. Case 1 is the R64Gfs*90 *TUBA4A* mutation case. Control = non-neurodegenerative control, PMI = *post-mortem* interval, RIN = RNA integrity number, IHC = immunohistochemistry, WB = western blot, n.a. = not applicable.

Case n°	Age	Sex	Diagnosis	Genetic mutation	Braak NFT stage	Aβ MTL phase	Braak LBD stage	PMI	RIN	IHC/WB/qPCR
**1**	60	m	FTLD-TDP Type C-like	*TUBA4A R64Gfs*90*	1	1	0	12h	4,5	IHC, WB, qPCR
**2**	69	f	FTLD-TDP Type C	no	1	0	0	24h	-	IHC, WB
**3**	62	f	FTLD-TDP Type C	no	0,5	0	0	12h	-	IHC, WB
**4**	79	m	FTLD-TDP Type B	no	1	0	0	24h	-	WB
**5**	64	f	FTLD-TDP Type A	*GRN IVS1+5G>C*	1	0	0	24h	-	WB
**6**	35	m	Control	n.a.	0	0	0	72h	-	WB
**7**	74	m	Control	n.a.	0	0	0	72h	-	WB
**8**	55	m	Control	n.a.	0	0	0	96h	-	WB
**9**	61	m	Control	n.a.	0	0	0	48h	6,6	WB, qPCR
**10**	54	m	Control	n.a.	1	0	0	24h	6,2	WB, qPCR
**11**	74	f	Control	n.a.	1	0	0	24h	6,5	IHC, qPCR

Supplementary Table S2. Overview of the central nervous system regions of the R64Gfs*90 *TUBA4A* mutation case positive (+) or negative (-) for pTDP-43 pathological lesions.

Frontal cortex	+	Thalamus	+
Precentral cortex	+	Basal nucleus of Meynert	-
Postcentral cortex	+	Amygdala	+
Parietal cortex	+	Midbrain - substantia nigra	-
Temporal cortex	+	Midbrain - red nucleus	-
Occipital cortex	+	Midbrain - formatio reticularis	+
Cingulate cortex	+	Midbrain - central gray matter	-
Entorhinal cortex	+	Midbrain - colliculi inferior	+
Hippocampus – CA region	+	Midbrain - raphe nuclei	-
Dentate gyrus	+	Pons - nuclei pontis	+
Subiculum	+	Pons - locus coeruleus	-
Insular cortex	+	Pons - parabrachial nucleus	-
Basal ganglia - caudate nucleus	+	Medulla - vagal nerve nucleus	-
Basal ganglia - claustrum	+	Medulla - inferior olivary nucleus	-
Basal ganglia - globus pallidus	+	Medulla - hypoglossal nucleus	-
Basal ganglia - putamen	+	Cerebellum	-
Hypothalamus	+	Cervical spinal cord	-
